# LoVE4MUM Mobile App to Prevent Postpartum Depression: Protocol for a Pilot Randomized Controlled Trial

**DOI:** 10.2196/63564

**Published:** 2025-01-27

**Authors:** Siti Sabrina Kamarudin, Idayu Badilla Idris, Shalisah Sharip, Norfazilah Ahmad

**Affiliations:** 1 Clinical Research Center Hospital Shah Alam, Institute for Clinical Research National Institute for Health Ministry of Health Malaysia Shah Alam Malaysia; 2 Department of Public Health Medicine Faculty of Medicine Universiti Kebangsaan Malaysia Cheras Malaysia; 3 Department of Psychiatry Faculty of Medicine Universiti Kebangsaan Malaysia Cheras Malaysia

**Keywords:** postpartum depression, mHealth intervention, mobile phone, prevention, self-guided, virtual engagement, engagement, maternal, protocol, randomized controlled trial, postpartum, depression, maternal, well-being, mobile health, preventive care, mobile app, mental health literacy, postpartum care

## Abstract

**Background:**

Postpartum depression remains a significant concern, posing substantial challenges to maternal well-being, infant health, and the mother-infant bond, particularly in the face of barriers to traditional support and interventions. Previous studies have shown that mobile health (mHealth) interventions offer an accessible means to facilitate early detection and management of mental health issues while at the same time promoting preventive care.

**Objective:**

This study aims to evaluate the effectiveness of the Leveraging on Virtual Engagement for Maternal Understanding & Mood-enhancement (LoVE4MUM) mobile app, which was developed based on the principles of cognitive behavioral therapy and psychoeducation and serves as an intervention to prevent postpartum depression.

**Methods:**

This single-blinded, pilot randomized controlled trial includes 64 mothers recruited from the postnatal ward and randomized using a 1:1 ratio to receive either postpartum care (treatment as usual) or postpartum care (treatment as usual) plus the self-guided LoVE4MUM mobile app. The primary outcome is the effectiveness of the mobile app at improving postpartum depression. Secondary outcomes are changes in the mental health literacy score and negative automatic thoughts, which are collected using a self-reported questionnaire.

**Results:**

Patient recruitment began on September 1, 2024. As of January 1, 2025, recruitment was successfully completed, with a total of 72 participants enrolled: 36 in the intervention group and 36 in the control group . The final results are anticipated to be available by March 2025, and publication is expected by the end of 2025.

**Conclusions:**

By examining the LoVE4MUM app alongside standard postpartum care, this pilot randomized controlled trial seeks to offer preliminary evidence on the potential of mHealth tools to improve maternal mental health as well as to reduce postpartum depression symptoms. The findings are expected to contribute to the future development of effective, accessible, and scalable interventions for mothers.

**Trial Registration:**

ClinicalTrials.gov NCT06366035; https://clinicaltrials.gov/study/NCT06366035

**International Registered Report Identifier (IRRID):**

PRR1-10.2196/63564

## Introduction

The global pooled prevalence of postpartum depression (PPD) was as high as 34% in a recent systematic review [1]. In Malaysia, the National Morbidity and Health Survey 2022 reported that 11.2% of mothers had depression [2] and an alarming 90% were unaware of their symptoms. Moreover, PPD results in detrimental consequences not only for the mother but also for the baby and their future bonding and relationship [3,4].

Meeting the increasing demand for mental health care is particularly challenging. These challenges encompass issues such as a lack of privacy, limited access to mental health experts, insufficient knowledge, and cultural stigma [5-8]. Consequently, mothers do not receive the necessary assistance that they require [5]. Mobile health (mHealth), a component of eHealth [9], has emerged as a crucial means to provide accessible health care solutions, especially with the advent of technologies like smartphones, web-based platforms, and text messaging [10,11]. These tools have become helpful for offering health care services and making health care more flexible, private, and readily available. This is particularly relevant in countries with high mobile internet penetration rates, such as Malaysia where rates of mobile internet coverage reached 128% in 2021 [12].

Interventions for depression range from pharmacotherapy to psychotherapy as well as lifestyle modifications [13]. Over the years, service delivery has evolved with the use of smartphone apps, web-based applications, and text messaging services to deliver therapeutic content [14,15]. Psychotherapies such as cognitive behavioral therapy (CBT) [16-18], mindfulness [19], and psychoeducation [20,21] using these technologies have previously been reported. A systematic review found that mHealth interventions can significantly improve depressive symptoms, highlighting their potential as accessible and cost-effective treatment options [22]. Additionally, more recent reviews concurred that mHealth interventions can effectively reduce PPD, as evidenced by improvements in Edinburgh Postnatal Depression Scale (EPDS) scores among these mothers [23,24]​​.

Women in the perinatal period are less likely to seek help for mental health issues than women at other life stages due to lower postpartum mental health literacy [25]. Mental health literacy has evolved from being defined merely as knowledge and beliefs about mental disorders to attitudes toward these disorders and efficacy in seeking help [26,27]. Kutcher et al [27] highlighted that poor mental health literacy leads to underutilization of mental health services, noting the critical role of help-seeking attitudes. mHealth platforms can boost mental health literacy by offering resources that heighten awareness of mental health issues, treatments, and self-care. For example, Chan et al [28] showed that mHealth is effective at enhancing mental health knowledge, attitudes, and behaviors, thus bridging the educational gap and reducing stigma. A systematic review emphasized the need for interventions to improve mental health literacy among perinatal women, due to a widespread lack of awareness about PPD symptoms, hesitance toward seeking professional help, a preference for informal support, and numerous other obstacles to accessing care, which are further compounded by marked stigma surrounding mental health issues [29].

Within the CBT framework, our thought processes are usually framed by our beliefs, which have a profound impact on how we feel and act in response to life's challenges [30]. Past experiences shape beliefs that can distort interpretations of events, leading to emotional disorders. In stress-triggering situations like the postpartum period, these beliefs trigger automatic negative thoughts and worsen depressive symptoms, underscoring the need to target these thoughts through depression management. By altering negative thought processes and maladaptive behaviors into more positive emotional outcomes, CBT offers improvements for depression management [31,32]. In addition, digital interventions, including exercises, thought diaries, and educational materials, facilitate the identification, confrontation, and modification of these negative cognitive patterns. A study demonstrated that smartphone-based CBT interventions could significantly reduce negative automatic thoughts and improve cognitive restructuring skills for users [33]. Mental health experts also advocate for short-term preventive CBT interventions, tailored for perinatal concerns, as a robust therapeutic strategy [34-36].

CBT’s traditional reliance on mental health professionals is challenged by cost and availability barriers. Due to health care personnel limitations, Mohammad-Alizadeh-Charandabi et al [37] highlighted the shortcomings of midwife-led phone interventions due to inconsistent quality and time constraints. In contrast, Niksalehi et al [38] effectively deployed structured automatic text messages with optional phone support, significantly enhancing depression scores and showcasing the potential of accessible, self-guided support. Complementing this, Carona et al [39] introduced a low-intensity, self-guided CBT program as an effective firstline intervention to help perinatal women, resulting in more improved depression scores in the intervention group than in the control group. Another study that used internet-based CBT [40] reported significant differences between the intervention and control groups.

It is noteworthy that the majority of studies on mHealth interventions were conducted in urban and academic settings in high-income countries [14]. Therefore, the generalizability of the outcomes is limited to the local population and might not apply to other settings such as Malaysia, which has a diverse cultural and religious background. Furthermore, these technologies frequently lack a user-centered design [41] and are primarily developed in western countries, which impacts their adaptability and sustainability in local contexts [14]. To culturally tailor appropriate mHealth interventions, meticulous development is required, engaging not only health care professionals to shape evidence-informed intervention priorities but also patients to enhance the effectiveness and widespread adoption of these interventions [42].

Initiatives in Malaysia include the TIARA MURNI project [43], which involves health care workers for its delivery. However, there is a notable absence of self-guided mental care solutions for mothers during the postpartum period especially in the Asian setting. This highlights an opportunity to improve mental health care access especially in areas with limited resources [38,44,45]. This study protocol intends to assess a user-centered, self-guided mental health app and compare it with standard postpartum care. It aims to determine the app’s preliminary effectiveness at improving depression and mental health literacy and addressing negative thoughts among mothers in the postpartum period.

## Methods

### Type and Design

This study is a single-blind, pilot randomized controlled trial with participants randomized using a 1:1 ratio.

### Study Location

The study includes postpartum mothers located in the postnatal ward of tertiary hospitals in the Selangor state of Malaysia. According to the Department of Statistics Malaysia, Selangor has the highest birth rate in the country, with a total of 95,211 live births in 2019 [46]. The National Morbidity and Health Survey 2022 survey indicated that urban mothers have a higher risk of depression. Considering Selangor’s birth statistics and status as a highly urbanized state, we considered it to be the ideal location for our research. We selected 2 hospitals from urban locations using a computerized random number generator.

### Ethics Approval and Ethical Considerations

Approval to conduct the study was obtained from the Medical Research and Ethics Committee, Ministry of Health Malays (ref number 24-00924-HPO, dated April 04, 2024). Written informed consent was obtained from all participants prior to their involvement in the study. Participants are fully informed of their right to opt out at any time without any consequences. All participant data are anonymized to ensure privacy and confidentiality. No identifiable information is collected nor stored during the study. Protective measures, including secure data storage, were implemented to safeguard participant information. Participants are compensated MYR 10 (US $2.15) for each questionnaire returned, to acknowledge their time and effort. Participation is entirely voluntary, without any form of coercion.

### Recruitment

Mothers were recruited from the obstetric wards by the investigators. Mothers received comprehensive information about the study, including its aims, procedures, risks, and benefits. Sufficient time was given to ensure informed consent was obtained in the ward. Those agreeing to participate were enrolled in the pilot randomized controlled trial, and reasons for refusal were documented.

Patients were screened for eligibility based on the study criteria. Mothers (1) scoring between 9 and 11 on the EPDS, (2) who had access to a smartphone, (3) who possessed home wireless internet or could access internet connectivity, (4) who planned to continue routine postpartum care in the government primary health facility, and (5) who were literate in both English and Malay were included in the study. Mothers (1) with a history of drug abuse; (2) who had been diagnosed with depressive illness in the current pregnancy; (3) who were undergoing treatment for depression, bipolar disorder, or any other psychiatric disease at the time of participation; and (5) whose infant experienced intrauterine death or death immediately after birth were excluded from the study.

### Randomization and Blinding

Participants were randomized (1:1) into 2 groups (ie, intervention and control groups). The researchers were blinded to the randomization process. Randomization was conducted by an independent assistant using a prearranged sealed opaque envelope. The envelopes contained a QR code linking to either a mobile app (the intervention group) or a WhatsApp communication channel (the control group). Both groups continue to receive treatment as usual (TAU) postpartum care.

### Intervention Group

The intervention group receives TAU postpartum care and the Leveraging on Virtual Engagement for Maternal Understanding & Mood-enhancement (LoVE4MUM) mobile app intervention.

The LoVE4MUM app represents an innovative self-guided approach to postpartum mental health care and offers a comprehensive, tailored intervention for maternal mental health during the postpartum period. It is available in English and the local Malay language. It was developed using a prior rigorous research process that included in-depth interviews with mothers who had symptoms of PPD and guidance from a multidisciplinary expert panel including clinical psychologists, psychiatrists, obstetricians, family health specialists, public health specialists, and epidemiologists with further input from previous literature [13,47-50]. This app-based intervention is grounded in psychoeducation, intrapersonal therapy, and CBT principles.

The app was validated by a panel of 4 experts. The content underwent detailed item-by-item validation to ensure its relevancy and clarity. In this face validation process, the majority of the items garnered unanimous approval, which was reflected in a content validity index and kappa coefficient of 1.00. Nevertheless, items 12 and 16 had content validity index and kappa scores <0.7, necessitating their revision to enhance relevancy within the module content. Additionally, item 18 was modified to provide better clarity. The expert panel reviewed the revisions and subsequently approved the finalized module. See Multimedia Appendix 1 for details of the validation process.

A linguistic expert translated the content into the Malay language to ensure that it was culturally and contextually appropriate. The app was developed on a self-developed website platform using a mobile phone interface. Snippets of the mobile app are available in Multimedia Appendix 2 and Multimedia Appendix 3.

The app is structured around 5 main modules that provide flexible and accessible support over a 6-week period. It does not require sequential module completion and allows for personalized pacing. Each module targets critical aspects of postpartum mental well-being and is delivered in several formats including video, infographics, notes, and worksheets. Each module takes approximately 20 minutes to 30 minutes to complete. Automated reminders incorporated into the app and linked to users’ emails encourage regular user engagement with app. The modules are described in Table 1.

**Table 1:** 

Module	Module description	Skills learned	Worksheet
Module 1: Learn to Love Yourself	This module emphasizes self-care, establishing goals, and navigating social comparisons while addressing typical challenges like managing expectations, hospital admissions, and handling baby feeding and care. It aims to empower mothers to prioritize their well-being.	Self-care practices, goal formulation, effective communication for seeking help, and strategies for tackling common issues	Setting goals and listing things you love
Module 2: Understanding Postpartum Depression	This module offers foundational knowledge about postpartum depression, aiming to increase awareness and reduce stigma.	Identifying postpartum depression, seeking help, treatment options, and assertive communication with partners and family	—^a^
Module 3: Addressing Unhelpful Thinking	This module integrates cognitive behavioral therapy (CBT) concepts, guiding mothers to recognize and question negative thinking and understand how thoughts influence behaviors.	Identifying unhelpful thinking habits and mechanisms to improve thought patterns	Encourages describing specific situations and working on improving the associated thoughts
Module 4: Mood Tracker	This module engages mothers in monitoring their mood and provides strategies for mood improvement.	Identifying emotions, identifying healthier responses, and learning how to perform deep breathing	—
Module 5: I Need Help Now	This module guides users in accessing immediate calming techniques, help, and essential information for assertive communication with health care providers.	Calm breathing, communication with health providers, and information on seeking help	—

^a^Not applicable.

All data in the mobile app are stored in a password-protected, secured data cloud handled by the app developer. To register to use the app, participants must create a password that is requested after every time they sign in. Only administrative personnel have access to the encrypted password data.

### Control Group

Both groups receive TAU postpartum care during the study. According to The Malaysian Perinatal Care Manual (fourth edition), at least 5 postnatal home visits and 1 clinic visit are conducted for normal cases, and the frequency increases for high-risk mothers and babies [51,52]. Routine postnatal care services include lactation consultation in the ward and postnatal visits by health care workers at home. Each postnatal check should assess the mother's overall, psychological, and emotional health, asking about any concerns, and evaluate the baby's well-being, feeding, and development to promptly identify any issues [53]. Participants are advised that they are free to withdraw from the study at any point for any reason.

### Measurements

The study uses a questionnaire delivered online (Google form) and divided into 3 outcome measurement tools.

### Primary Outcome Measurement

The EPDS is a 10-item questionnaire extensively used to assess postnatal depression in both international and local contexts. It uses a 4-point Likert scale for responses, with higher scores indicating greater depressive symptoms. The scale has been translated into the Malay language [54], where a score of 11.5 was the optimum cut-off for 72.7% sensitivity, 95% specificity, and a positive predictive value of 80%. In our study, participants with an EPDS score ≥12 are categorized as having postpartum depressive symptoms.

### Secondary Outcome Measurements

#### Postpartum Depression Literacy Scale (PoDLiS)

The Postpartum Depression Literacy Scale (PoDLiS) is a 31-item, self-administered questionnaire developed by Mirsalimi et al [55] to assess the understanding of PPD. It evaluates 7 attributes of PPD literacy, including recognition, knowledge of risk factors and causes, self-care activities, available professional help, attitudes toward recognition and help-seeking, and information sources. Responses are scored on a 5-point scale, with attribute scores calculated by averaging related item scores. The Malay version of PoDLiS has acceptable reliability, with a Cronbach α of 0.73.

#### Automatic Thought Questionnaire (ATQ)

The Automatic Thought Questionnaire (ATQ), which is available in the Malay language [56], assesses the frequency of negative automatic thoughts linked to depression and anxiety. This 17-item scale asks respondents to rate the occurrence of specific negative thoughts over the past week on a scale from 1 to 5. Higher total scores indicate more frequent negative thinking. The scale’s internal consistency ranges from 0.83 to 0.93 [56]. The total score ranges from 17 to 85, with a higher score reflecting a higher frequency of negative thinking.

Data are conducted at baseline, 1 week postpartum, and 6 weeks postpartum using the Google form, which is delivered via either the mobile app or a Whatsapp number. The self-reported survey takes approximately 15 minutes to 20 minutes to complete. All answers are automatically storied in the Google drive, and only the research team has access to the data. The schedule of enrollment, interventions, and assessments is summarized in Multimedia Appendix 4 and Multimedia Appendix 5.

### Sample Size

Based on the recommendations of Whitehead et al [57], for a pilot trial with 90% power and a 2-sided significance level of 5%, the suggested sample size per treatment arm based on previous literature with a medium effect size (0.43) [58] is 25. Hence, a total of 72 participants (36 participants per arm) was required after taking into consideration a 30% nonresponse rate.

### Statistical Analysis

Data will be analyzed using SPSS version 26, and significance is set at *P*<.05. Intention-to-treat analysis will be used. Categorical variables will be presented as the frequency and percent values, while continuous data will be presented as mean (SD). Baseline between-group comparisons will be conducted using Pearson chi square and Fisher exact tests for categorical data. Meanwhile, independent *t* tests will be used for continuous data. Repeated measures ANOVA will be used for within-group (time effect) and between-group (treatment effect) comparisons at 6 weeks. The mean difference and its corresponding 95% CI will be reported, with the significant level set at *P*<.05.

### Risk Management

All participants were informed that the intervention serves as a supplementary self-help program and does not replace professional health care services. They are strongly encouraged to seek advice from their doctors or general practitioners regarding any concerns related to their mental or general health. Participants who experience symptoms of depression during the pilot trial are urged to promptly consult health care professionals and inform the research team of their condition. These measures allow for the appropriate management of any adverse effects and ensure the well-being of participants throughout the study.

For those screened and found ineligible for the trial, comprehensive support information was provided through a brochure containing information on PPD, immediate assistance, and emergency contact details. These individuals are also advised to consult their health care providers if they have concerns about their mental health. Specifically, mothers with an EPDS score ≥12 were excluded and referred to specialists for further evaluation and intervention in line with the Malaysian guideline for managing PPD.

Moreover, all participants continue to receive postpartum care by attending health care professionals at the Malaysia’s Ministry of Health facilities, with care adhering to the standards set forth in the Perinatal Care Manual (fourth edition) [51].

## Results

Participant recruitment began on September 1, 2024. As of January 1, 2025, recruitment was complete, resulting in a total of 72 participants enrolled: 36 in the intervention group and 36 in the control group. Data analysis and results are anticipated to be available by March 2025. The trial results are expected to be published by the end of 2025.

## Discussion

### Overview

PPD is influenced by a combination of intrapersonal, environmental, and physical factors that necessitates multifaceted interventions. The LoVE4MUM app represents a potential intervention for PPD prevention and uses an innovative approach that integrates the complexity of PPD with the unique cultural and societal contexts of Malaysia. The app combines psychosocial interventions, including CBT components, to address negative automatic thoughts and improve mental health literacy through engaging, educational content. The authors anticipate that participants receiving the LoVE4MUM app will demonstrate improvements in depressive symptoms, as measured by EPDS, compared with those receiving only standard postpartum care. Previous studies highlight the potential of mobile apps to improve mental health in perinatal populations [59]. Several reviews on mHealth interventions have revealed significant reductions in EPDS scores among postpartum mothers using mobile apps ​ [23,60-62]. The LoVE4MUM app is designed to provide culturally relevant content. It also considers the barriers postpartum mothers in Malaysia face, such as limited access to face-to-face care and social stigma surrounding mental health.

The COVID-19 pandemic amplified the global prevalence of PPD, with rates within middle-income countries increasing from 20.8% before the pandemic to 34% during the pandemic [1,63]​. This surge emphasizes the urgent need for remote health care solutions. The LoVE4MUM app leverages this shift by offering a self-guided platform that empowers mothers to engage in self-care from the convenience of their smartphones, circumventing the limitations of in-person care.

Lin et al [64] demonstrated that digital interventions promoting psychological empowerment and hedonic well-being are effective at driving these behavioral changes. In line with these findings, the LoVE4MM app encourages mothers to engage in activities that enhance their well-being while offering tools for cognitive restructuring. This approach is particularly crucial in a cultural context like Malaysia, where mental health stigma may prevent mothers from seeking professional therapy. Compared with the control group, we expect that mothers using the app will have improved EPDS scores; better scores for mental health literacy, as measured by the PoDLiS tool; and reductions in automatic negative thoughts.

Additionally, mHealth interventions alleviate depressive symptoms by improving social support [65,66]. Enhancing environmental support through assertive communication enables mothers to seek effective help from spouses and family members, reducing stress and improving mental health [67-69]. Similar to the CareMom app, which demonstrated a significant reduction in depressive symptoms through communication exercises, assertive requests within the CBT framework in the LoVE4MUM app are expected to improve emotional well-being during the postpartum period [70].

Anto et al [71] and Hartnup et al [72] showed that postpartum mothers often experience social risk and pressure through social media, which can exacerbate stress by promoting unrealistic comparisons and idealized identities of motherhood. Many mothers feel pressured to project a “perfect” image, leading to feelings of inadequacy and increased mental strain. By addressing these concerns, the LoVE4MUM app emphasizes the importance of self-compassion and reinforces a supportive, nonjudgmental digital environment, encouraging mothers to focus on doing their best rather than striving for perfection, which is crucial for improving mental well-being in this vulnerable period.

The strength of this pilot trial lies in its integration of validated measurement tools—EPDS, PoDLIiS, and ATQ—which offer a comprehensive view of maternal mental health. We expect the LoVE4MUM app's CBT-based interventions to demonstrate the success seen in similar applications, such as the CareMom, MumMoodBoster, Sunnyside, and Mother and Babies Course apps, which have successfully used structured exercises and personalized feedback to reduce depressive symptoms [18,70,73-76].

Nevertheless, certain challenges remain. First, the reliance on self-reported measures may introduce bias, as participants may underreport symptoms due to stigma or discomfort. Second, as demonstrated with other mHealth interventions like the Happy Mother app, user engagement can be inconsistent, and low adherence rates may diminish the overall effectiveness of the intervention​ [77]. To address this, the LoVE4MUM app incorporates strategies to enhance user engagement, such as push notifications, scripts, and personalized feedback. Another critical aspect to consider is the influence of cultural factors on app engagement and perception, which may affect how Malaysian mothers interact with the app. Ensuring that the app’s content is culturally sensitive and tackles common misconceptions about PPD will be essential in maximizing its impact. Moreover, as a pilot trial, the sample size may not allow for definitive conclusions, and the study’s findings should be interpreted as preliminary evidence of the app's potential.

### Conclusion

The LoVE4MUM app represents a promising, scalable solution to address PPD among Malaysian mothers. If successful, this app could serve as a model for culturally adapted digital interventions that empower mothers to manage their mental health, ultimately reducing the prevalence and impact of PPD. Future studies will be necessary to further refine the app’s features and evaluate its long-term impact on maternal mental health outcomes.

## Appendix

Multimedia Appendix 1 Expert validation of LoVE4MUM module content.

Multimedia Appendix 2 Snippets from LoVE4MUM mobile app login interface.

Multimedia Appendix 3 Snippets from LoVE4MUM mobile app module Postpartum Depression.

Multimedia Appendix 4 Patient visit schedule.

Multimedia Appendix 5 Consolidated standard of reporting trials flow diagram of the LoVE4MUM pilot trial.

## Abbreviations

ATQ: Automatic Thought Questionnaire
CBT: cognitive behavioral therapy
EPDS: Edinburgh Postpartum Depression Scale
LoVE4MUM: Leveraging on Virtual Engagement for Maternal Understanding & Mood-enhancement
mHealth: mobile health
PoDLiS: Postpartum Depression Literacy Scale
PPD: postpartum depression
TAU: treatment as usual

## Data Availability

The data sets generated or analyzed during this study are available from the corresponding author on reasonable request.
