# Supplementary figures and images for: LoVE4MUM Mobile App to Prevent Postpartum Depression: Protocol for a Pilot Randomized Controlled Trial
Source: JMIR Res Protoc. 2025 Jan 27;14:e63564. doi: 10.2196/63564 (PMC11811676; doi:10.2196/63564)

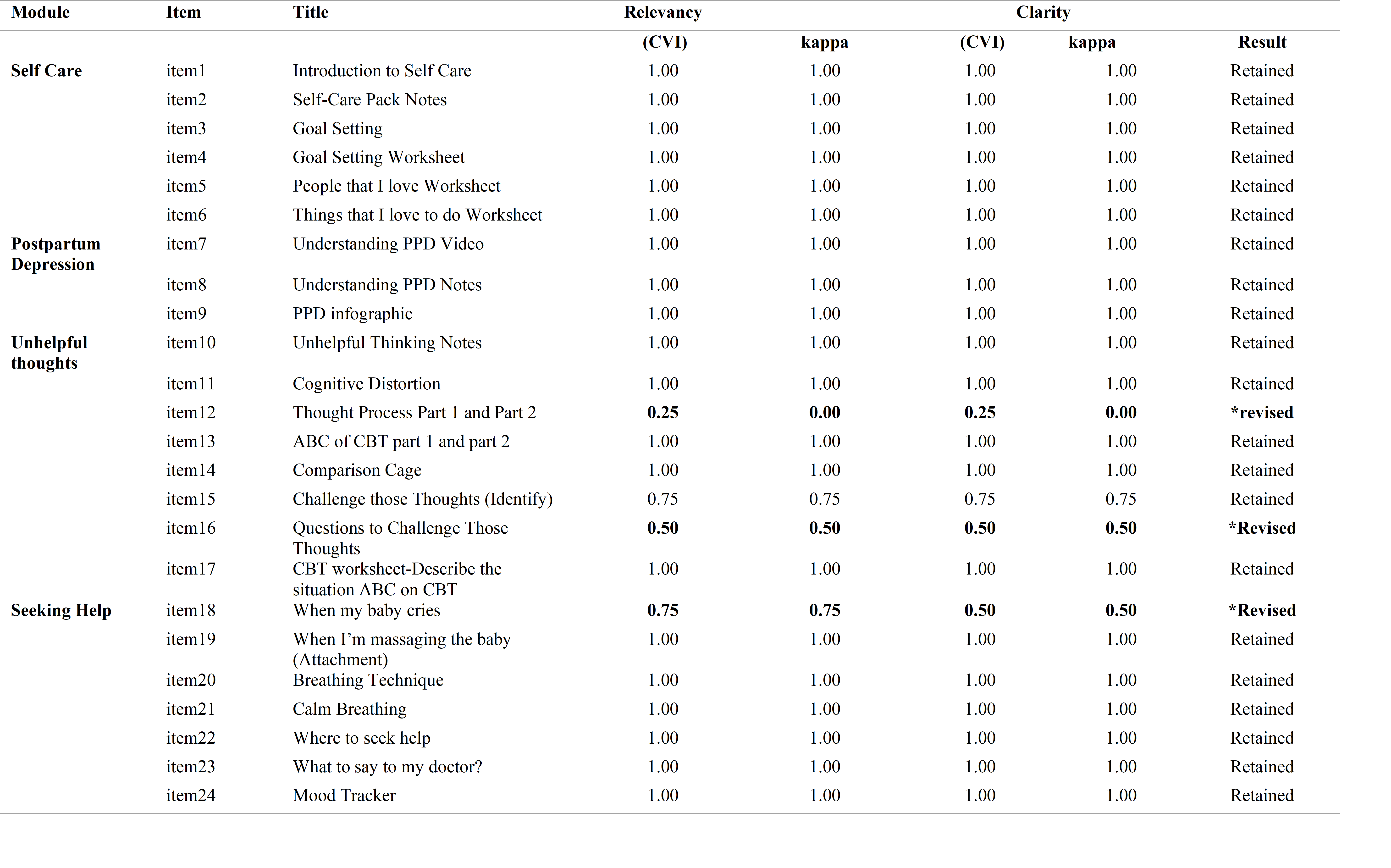

Supplement: Multimedia Appendix 1 [file resprot_v14i1e63564_app1.png]

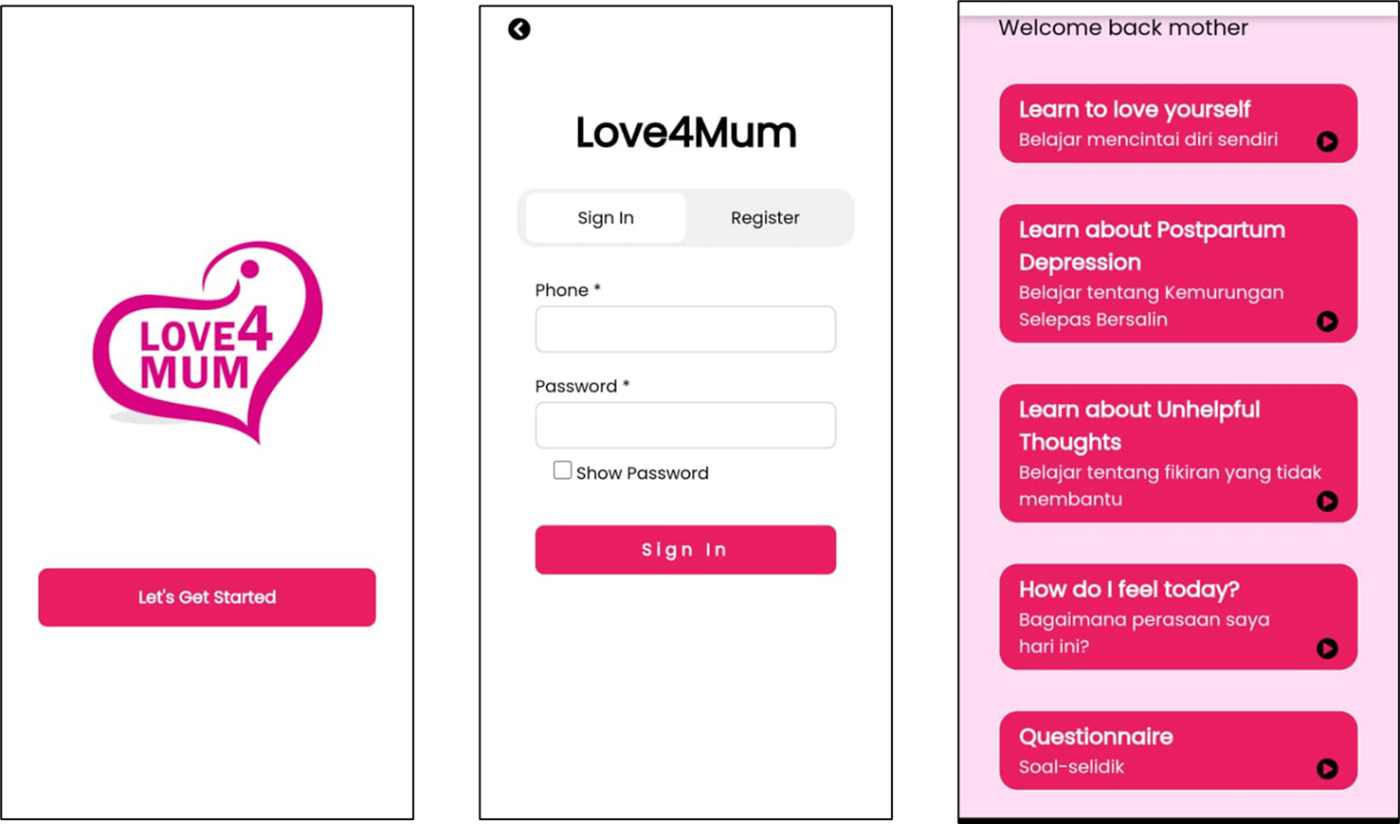

Supplement: Multimedia Appendix 2 [file resprot_v14i1e63564_app2.png]

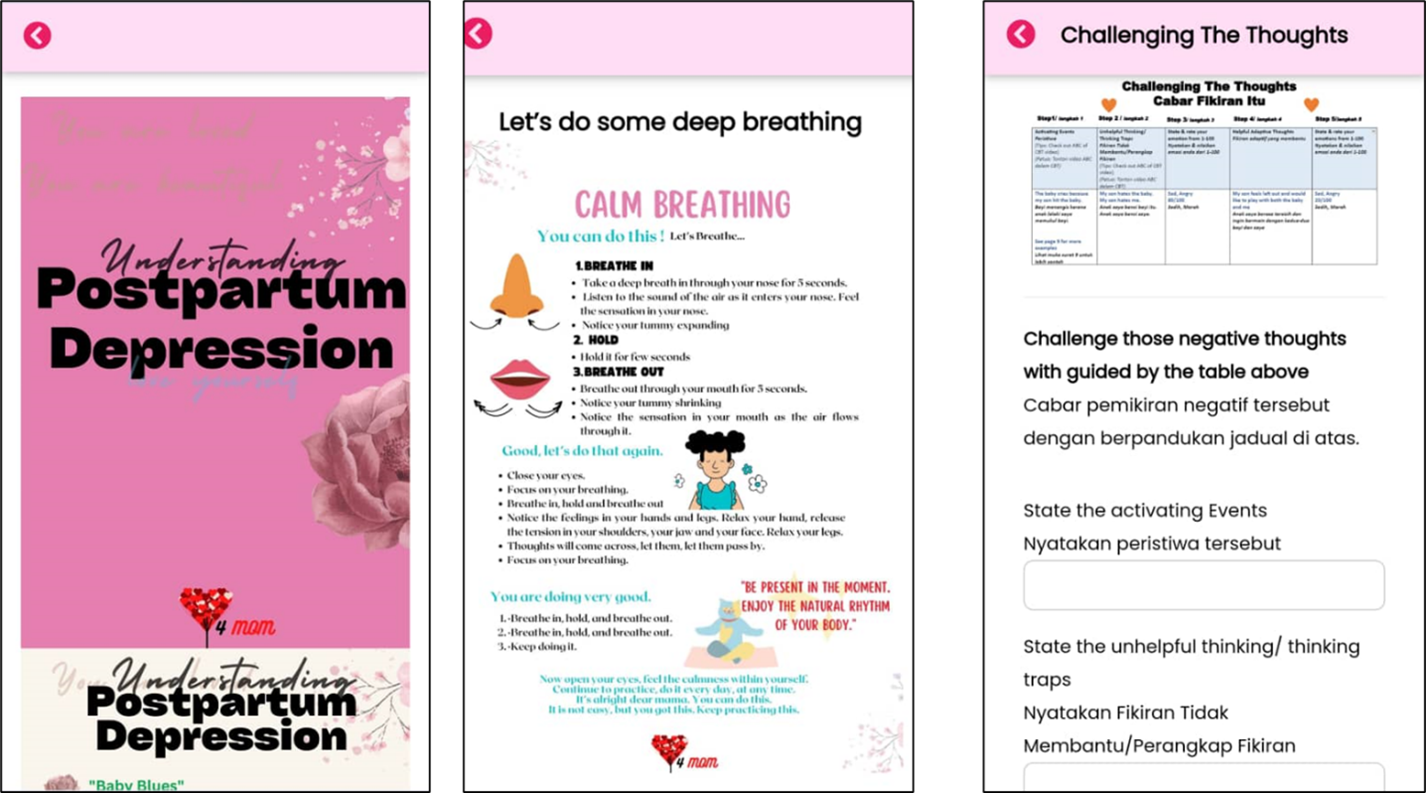

Supplement: Multimedia Appendix 3 [file resprot_v14i1e63564_app3.png]

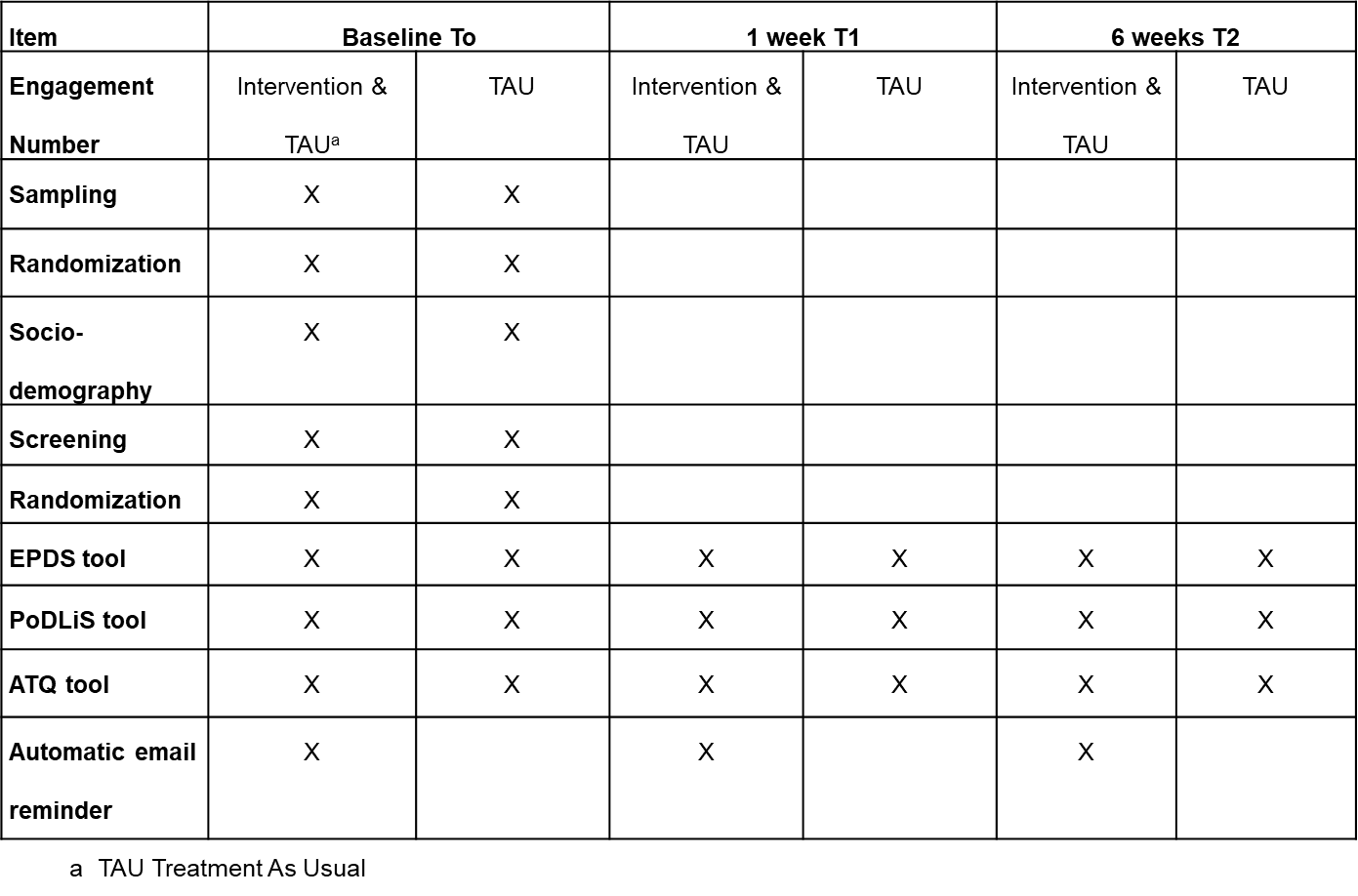

Supplement: Multimedia Appendix 4 [file resprot_v14i1e63564_app4.png]

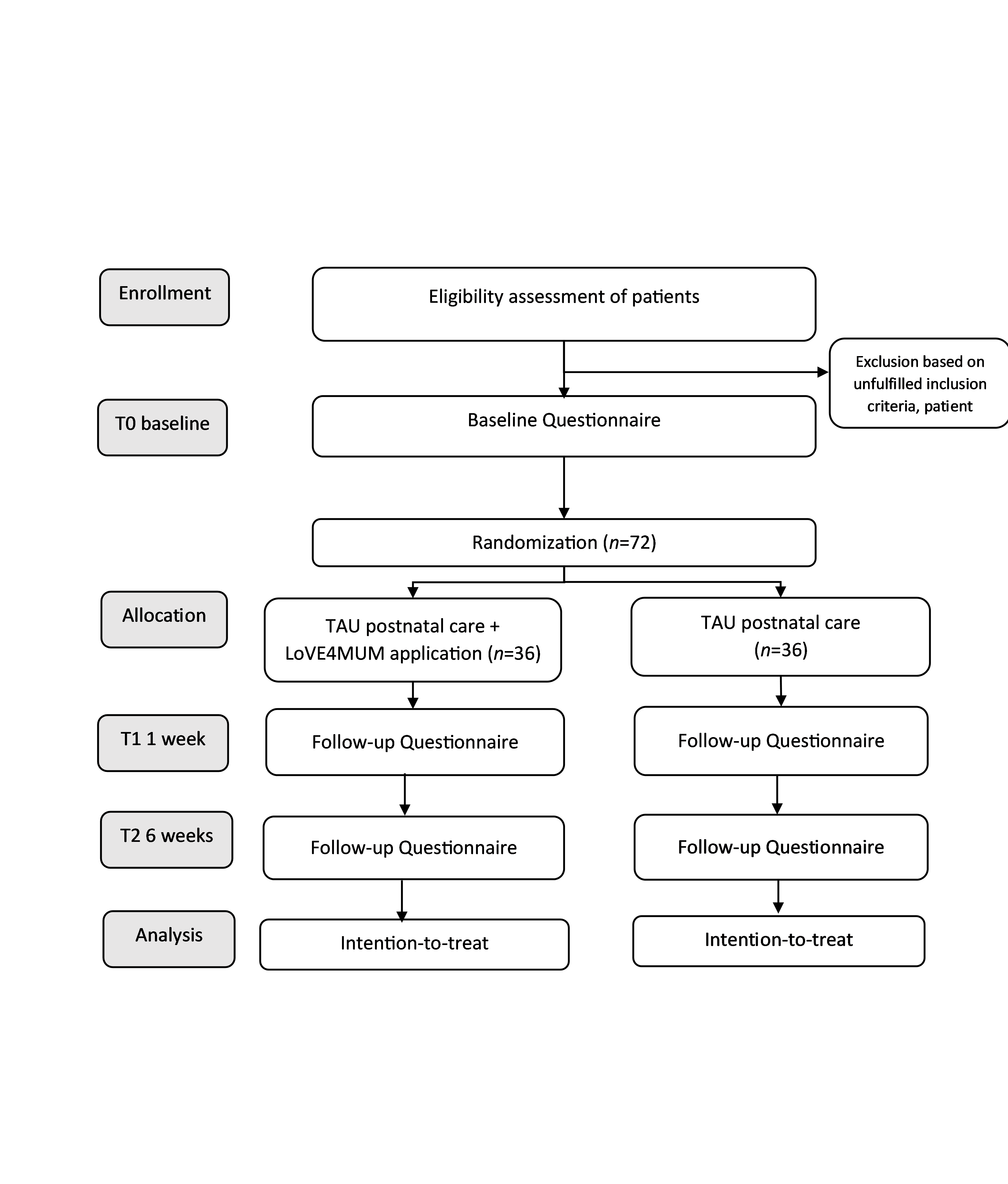

Supplement: Multimedia Appendix 5 [file resprot_v14i1e63564_app5.png]
